# Obstructive Sleep Apnea: A View from the Back Door

**DOI:** 10.3390/medicina56050208

**Published:** 2020-04-25

**Authors:** Silvano Dragonieri, Andras Bikov

**Affiliations:** 1 Department of Respiratory Diseases, University of Bari, 70124 Bari, Italy; 2 North West Lung Centre, Manchester University NHS Foundation Trust, Manchester M239LT, UK; 3 Division of Infection, Immunity and Respiratory Medicine, University of Manchester, Manchester M239LT, UK

**Keywords:** obstructive sleep apnea, sleep disordered breathing, cardiovascular comorbidities, biomarkers, inflammation, volatile organic compounds, accident risk, non-communicable diseases, risk assessment

## Abstract

Obstructive sleep apnea (OSA) is a common disease that may affect up to 50% of the adult population and whose incidence continues to rise, as well as its health and socio-economic burden. OSA is a well-known risk factor for motor vehicles accidents and decline in work performance and it is frequently accompanied by cardiovascular diseases. The aim of this Special Issue is to focus on the characteristics of OSA in special populations which are less frequently investigated. In this regard, seven groups of experts in the field of sleep medicine gave their contribution in the realization of noteworthy manuscripts which will support all physicians in improving their understanding of OSA with the latest knowledge about its epidemiology, pathophysiology and comorbidities in special populations, which will serve as a basis for future research.

## Introduction

Obstructive sleep apnea (OSA) is a common disease that may affect up to 50% of the adult population [1]. These percentages are comparable to arterial hypertension [2], and even higher than in diabetes mellitus [3]. Although the exact prevalence in different communities is still unknown, the incidence of OSA continues to rise, as well as its health and socio-economic burden [4]. This Special Issue focuses on the characteristics of OSA in special populations which are less frequently investigated.

OSA is a well-known risk factor for motor vehicles accidents and decline in work performance [5,6]. Alexandropolou et al. concluded that OSA affects around 20% of the Greek nurses and 8% of the nurses have OSA with excessive daytime sleepiness [7]. Celikhisar et al. studied 965 heavy equipment operators in Turkey and found that around 7% of them had OSA [8]. More importantly, the severity of OSA was directly related to the number of work-related accidents [8].

Despite the increasing awareness of OSA and its consequences, most of the patients with OSA remain undiagnosed and untreated [9]. Data on OSA prevalence mainly originate from high-income countries with good healthcare access [4]. In contrast, low- or middle-income countries are less-represented in epidemiological studies. Mathiyalagen et al. screened a population of patients attending non-communicable disease clinics in a rural health training center in South India and reported a 25.8% incidence of OSA [10].

Cardiovascular diseases frequently accompany OSA [11]. Chronic intermittent hypoxia in OSA leads to airway inflammation [12] which can be analyzed in exhaled breath samples [13]. In this issue, Finamore et al. provide a comprehensive summary on the current knowledge of exhaled breath analysis in OSA [14]. Airway inflammation, together with intermittent hypoxia and surges in the sympathetic activity, induce systemic inflammation [15] which could be a potential link to cardiovascular diseases in OSA. The soluble urokinase type plasminogen activator receptor (suPAR) is a promising biomarker of cardiovascular disease [16]. However, Bocskei et al. reported unaltered suPAR levels in OSA [17]. Despite the relationship between cardiovascular disease and OSA, little is known about the characteristics of obstructive sleep apnea in special subgroups of patients. Ardelean et al. studied 143 patients with heart failure and OSA [18]. They concluded that patients with mid-range ejection fraction (40%–49%) are characterized by a different profile of comorbidities compared to low and preserved ejection fraction subgroups [18]. Finally, in their excellent study, Zota el al. concluded that OSA is related to exercise limitation which is improved after continuous positive airway treatment [19].

Taken together, these studies will support all physicians in improving their understanding of OSA with the latest knowledge about its epidemiology, pathophysiology and comorbidities in special populations, which will serve as a basis for future research.
